# Anorectal leiomyoma with GLUT1 overexpression mimicking malignancy on FDG-PET/CT

**DOI:** 10.1093/jscr/rjac101

**Published:** 2022-05-24

**Authors:** Fuminori Teraishi, Kunitoshi Shigeyasu, Shunsuke Kagawa, Toshiyoshi Fujiwara

**Affiliations:** Department of Gastroenterological Surgery, Okayama University Graduate School of Medicine, Dentistry and Pharmaceutical Sciences, Okayama, Japan; Department of Minimally Invasive Therapy Center, Okayama University Hospital, Okayama, Japan; Department of Gastroenterological Surgery, Okayama University Graduate School of Medicine, Dentistry and Pharmaceutical Sciences, Okayama, Japan; Department of Minimally Invasive Therapy Center, Okayama University Hospital, Okayama, Japan; Department of Gastroenterological Surgery, Okayama University Graduate School of Medicine, Dentistry and Pharmaceutical Sciences, Okayama, Japan; Department of Minimally Invasive Therapy Center, Okayama University Hospital, Okayama, Japan; Department of Gastroenterological Surgery, Okayama University Graduate School of Medicine, Dentistry and Pharmaceutical Sciences, Okayama, Japan; Department of Minimally Invasive Therapy Center, Okayama University Hospital, Okayama, Japan

## Abstract

A 43-year-old female underwent pelvic magnetic resonance imaging for uterine myoma that incidentally revealed a 4.6 × 2.8 cm soft tissue mass in the anorectal region. Rectal endoscopy showed a submucosal tumor just above the anal canal. Fluorodeoxyglucose-positron emission tomography/computed tomography (FDG-PET/CT) revealed an anorectal tumor with very high FDG uptake. Aspiration cytology and needle biopsy were inconclusive, and the patient underwent trans-perineal tumor resection. The excised tumor was a 4.6 × 3.5 × 2.7 cm gray–white bifurcated nodular tumor. Light microscopy revealed fenestrated growth of poorly dysmorphic short spindle-shaped cells with eosinophilic sporophytes. Immunohistochemical staining was positive for αSMA and desmin, negative for CD117 (KIT) and S100, and the patient was diagnosed with benign leiomyoma. Tumor cells were also positive for glucose transporter-1 (GLUT1) immunohistochemically. It is important to keep in mind that FDG-PET/CT may show false-positive results even in benign anal leiomyoma for various reasons, including GLUT1 overexpression.

## INTRODUCTION

Leiomyomas of the gastrointestinal tract occur most commonly in the esophagus and rectum. Rectal leiomyoma presents as small polypoid lesions arising from the muscularis mucosae [1]. Although the external anal sphincter itself is a striated muscle, leiomyoma can also occur in the perianal region; i.e. in the anal fossa of the intestine [2]. Fluorodeoxyglucose-positron emission tomography/computed tomography (FDG-PET/CT) is widely used to differentiate between benign and malignant tumors and to detect distant metastasis and recurrence [3]. Here, we report a rare case of FDG-PET/CT-positive anorectal benign leiomyoma that was associated with glucose transporter-1 (GLUT1) overexpression.

## CASE REPORT

**Figure 1:**
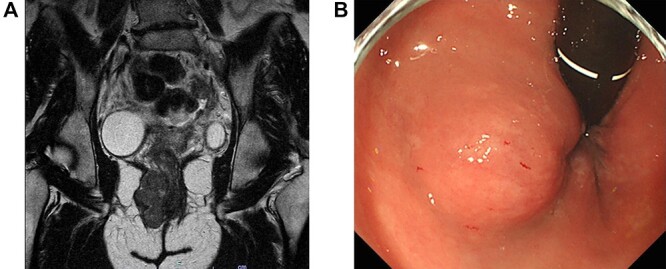
(**A**) Magnetic resonance imaging of the pelvis (sagittal section) demonstrates a 4.6 × 2.8-cm tumor in the right perineal area. (**B**) Proctoscopy reveals a submucosal tumor near the right wall of the anal canal.

**Figure 2:**
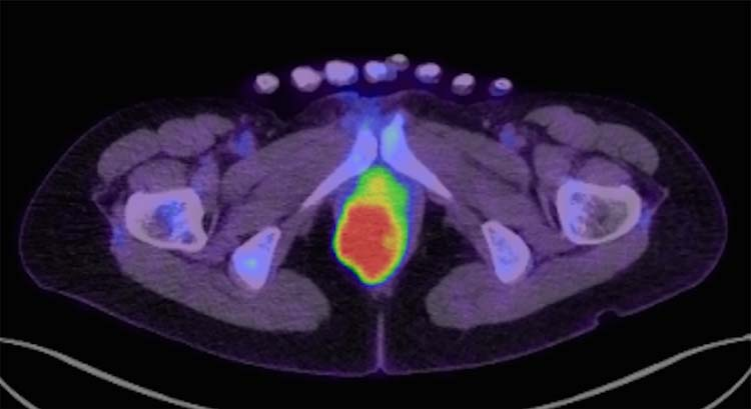
FDG-PET/CT (transverse section) show FDG accumulation in a mass with maximum standardized uptake of 17.9.

A 43-year-old female underwent pelvic magnetic resonance imaging (MRI) for uterine myoma that incidentally revealed a 4.6 × 2.8 cm soft tissue mass in the right perineal area (Fig. 1A). Rectal endoscopy showed a submucosal tumor just above the anal canal (Fig. 1B). FDG-PET/CT scan revealed an anorectal tumor with very high ^18^F-FDG uptake (SUVmax = 17.9; Fig. 2). As aspiration cytology and needle biopsy were inconclusive, we planned to perform trans-perianal tumor resection.

**Figure 3:**
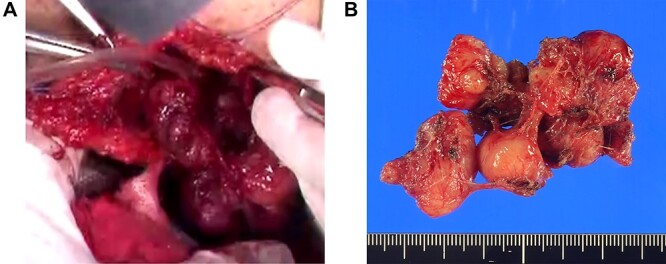
(**A**) Extracapsular dissection was performed and sphincter fibers were repaired with absorbable thread. (**B**) A gray–white bifurcated nodular tumor of size 4.6 × 3.5 × 2.7 cm was excised.

**Figure 4:**
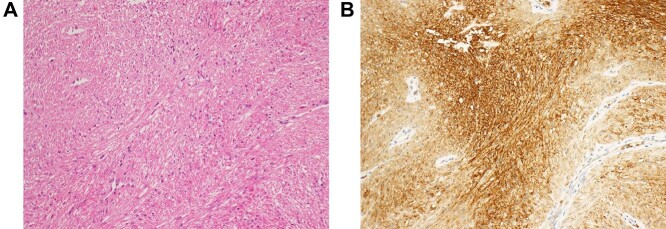
(**A**) Histopathological examination reveals fenestrated growth of poorly dysmorphic short spindle-shaped cells. (**B**) Immunohistochemical staining shows that the tumor cells are positive for GLUT-1.

With the patient in the jackknife position, a 7-cm longitudinal incision was made on the right margin of the anus, the index finger was inserted into the rectum and the tumor was palpated to confirm. The tumor was covered with a thin capsule and sphincter fibers were stretched over the surface of the lesion. Complete extracapsular dissection was performed and the sphincter fibers were repaired with absorbable thread (Fig. 3A). The excised gray–white tumor was bifurcated and nodular, of size 4.6 × 3.5 × 2.7 cm (Fig. 3B). Light microscopy revealed fenestrated growth of poorly dysmorphic short spindle-shaped cells with eosinophilic sporophytes, but no cell atypia or mitoses (Fig. 4A). Immunohistochemical staining was positive for αSMA and desmin, negative for CD117 (KIT) and S100, and the patient was therefore diagnosed with benign leiomyoma. Tumor cells were also positive for GLUT1 immunohistochemically (Fig. 4B). The patient was discharged on the eighth post-operative day without no complications such as sphincter insufficiency. One year after surgery, the patient is under outpatient follow-up without recurrence.

## DISCUSSION

Leiomyoma is a benign tumor that arises from smooth muscle cells. Gastrointestinal leiomyomas are most common in the esophagus and rectum. Anorectal leiomyomas are rare, occurring in one out of every 2000 or 3000 rectal tumors [4]. There are no specific symptoms, and the tumor is often discovered incidentally during digital examination or colonoscopy. In diagnosis, imaging tests such as MRI and endoscopic ultrasonography show no specific findings, and qualitative diagnosis is difficult. Even when leiomyosarcoma is diagnosed histologically, local recurrence or metastasis is seen in 31% of cases after surgery [5]. Although leiomyoma has a good prognosis, the 5-year survival rate after extended surgery for leiomyosarcoma is poor, ranging from 20 to 25% [6]. Therefore, the crucial issue in the diagnosis and treatment of gastrointestinal leiomyoma is the difficulty in determining whether the tumor is benign or malignant.

In recent years, FDG-PET/CT has been widely used for the differential diagnosis of benign and malignant neoplastic lesions. FDG-PET/CT is based on the principle that FDG, like glucose, is taken up into cells by the glucose transporter (GLUT) on the cell membrane and phosphorylated by hexokinase to FDG-6-phosphate [7]. The GLUT on the cell membrane takes up FDG as well as glucose and phosphorylates it to FDG-6-phosphate by hexokinase. It is known that overexpression of GLUT isozymes and increased hexokinase activity are observed in tumor cells [8, 9].

Rectal leiomyoma is a benign tumor with low mitotic activity and does not usually show increased accumulation on FDG-PET/CT. Meanwhile, leiomyosarcoma often shows mitotic activity and FDG accumulation has been reported in many cases [10, 11]. We found only one previous case of rectal leiomyoma with FDG hyperaccumulation on FDG-PET/CT [12]. In the present case, the primary cause of FDG accumulation in the tumor might be overexpression of GLUT-1, and this is the first study to report benign anorectal leiomyoma demonstrating FDG accumulation on FDG-PET/CT with GLUT1 overexpression. Regarding treatment, the possibility of malignancy could not be ruled out because of the increased FDG accumulation on FDG-PET/CT, and surgical resection was therefore performed. We developed a two-stage surgical treatment plan: a less invasive local excision to preserve the sphincter, followed by additional radical resection in the event of malignant findings after pathological examination of the resected specimen. Less invasive surgery such as trans-sacral or trans-anal local excision is often performed for benign anorectal tumors; however, even if the sphincter muscle can be preserved, there are many problems in terms of decreased QOL, including fecal incontinence. In the present case, the tumor was located on the right wall of the rectum, and we decided to use the trans-perianal approach because the surgical field was better developed, the distance to the tumor was shorter, and contamination of the surgical field could be avoided compared with the trans-anal approach. In cases where surgical treatment is likely to cause significant disturbance to the patient’s quality of life (such as sphincter function), a less invasive local excision may be an option, followed by additional excision if necessary after examination of the resected specimen. Moreover, we should consider the differential diagnosis for anorectal neoplasms taking into account that benign anorectal leiomyomas are potential causes of false-positive FDG-PET/CT.

## CONCLUSION

Anorectal leiomyoma is a rare benign lesion. We report a 42-year-old female with anorectal leiomyoma that was suspected to be malignant based on abnormal accumulation on FDG-PET/CT, which was associated with GLUT1 overexpression.
